# Supplementary material for: Mechanically Enhanced Detoxification of Chemical Warfare Agent Simulants by a Two-Dimensional Piezoresponsive Metal–Organic Framework
Source: Nanomaterials (Basel). 2024 Mar 22;14(7):559. doi: 10.3390/nano14070559 (PMC11013765; doi:10.3390/nano14070559)
Supplement: Supplementary file 1 [file nanomaterials-14-00559-s001.zip › nanomaterials-2884677-supplementary.pdf]

Article

Mechanically Enhanced Detoxification of Chemical Warfare Agent Simulants by a Two-Dimensional Piezoresponsive Metal–Organic Framework

Yuyang Liu †, Shiyin Zhao †, Yujiao Li, Jian Huang, Xuheng Yang, Jianfang Wang * and Cheng-an Tao *

College of Science, National University of Defense Technology, Changsha 430083, China; liuyuyang0412@163.com (Y.L.); zhaoshiyin21@nudt.edu.cn (S.Z.); liyujiao15@nudt.edu.cn (Y.L.); huangjian2015@nudt.edu.cn (J.H.); ouyangxuheng@163.com (X.Y.)

* Correspondence: jianfangwang@nudt.edu.cn (J.W.); chengantao@nudt.edu.cn (C.-a.T.)

† These authors contributed equally to this work.

This supporting information contained 3 figures (Figure S1–S3).

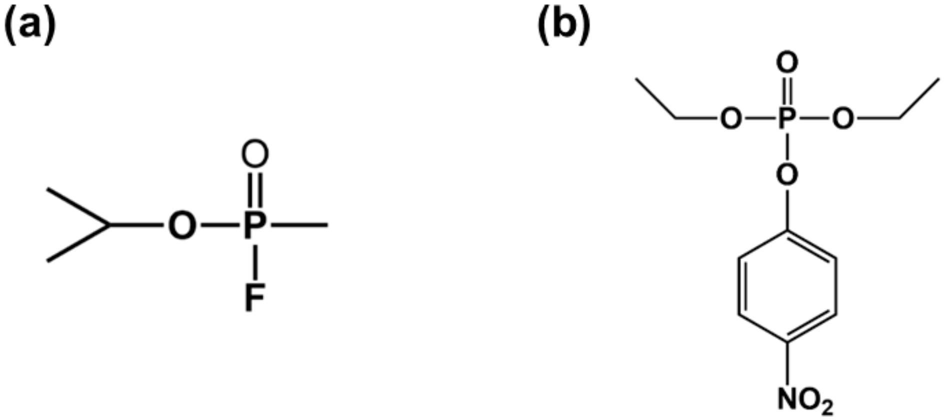

Figure S1. The chemical structure of (a) Sarin and (b) simulant DMNP.

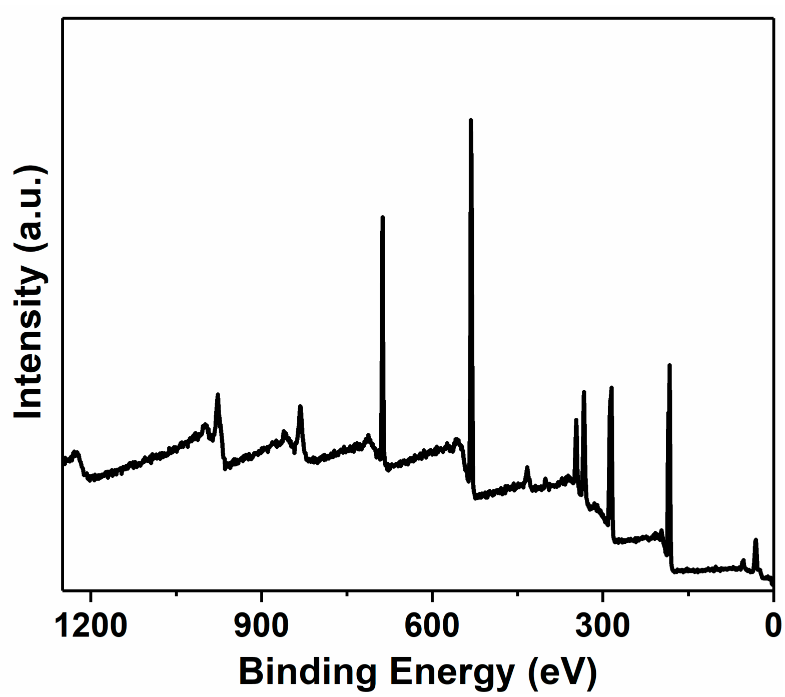

Figure S2. The XPS survey spectra of UiO-66-F_4.

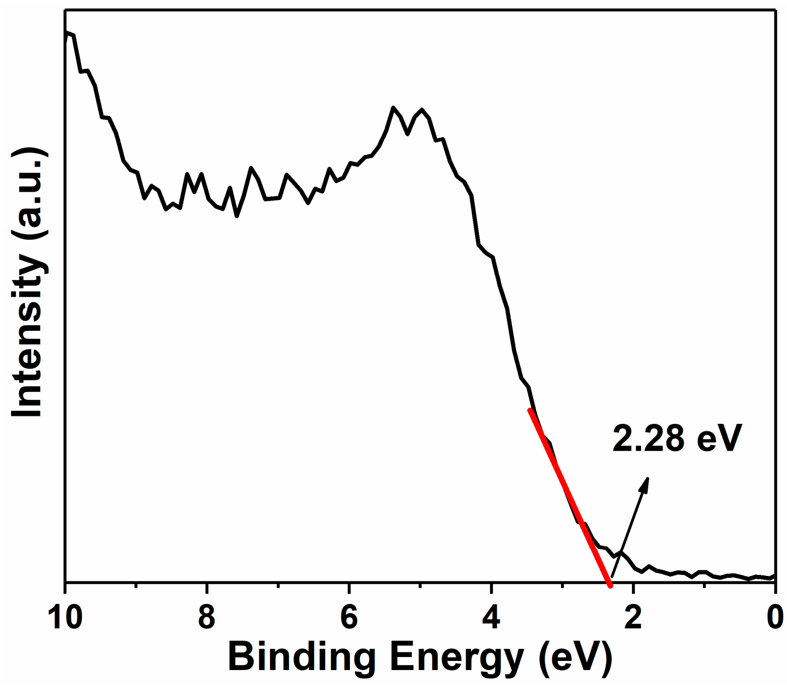

Figure S3. The valence XPS spectra of UiO-66-F_4.
